# Translational read-through of the RP2 Arg120stop mutation in patient iPSC-derived retinal pigment epithelium cells

**DOI:** 10.1093/hmg/ddu509

**Published:** 2014-10-06

**Authors:** Nele Schwarz, Amanda-Jayne Carr, Amelia Lane, Fabian Moeller, Li Li Chen, Mònica Aguilà, Britta Nommiste, Manickam N. Muthiah, Naheed Kanuga, Uwe Wolfrum, Kerstin Nagel-Wolfrum, Lyndon da Cruz, Peter J. Coffey, Michael E. Cheetham, Alison J. Hardcastle

**Affiliations:** 1 UCL Institute of Ophthalmology, 11-43 Bath Street, London EC1V 9EL, UK,; 2 Johannes Gutenberg-University Muellerweg 6, 55099 Mainz, Germany and; 3 Moorfields Eye Hospital, 162 City Road, London EC1V 2PD, UK

## Abstract

Mutations in the *RP2* gene lead to a severe form of X-linked retinitis pigmentosa. *RP2* patients frequently present with nonsense mutations and no treatments are currently available to restore RP2 function. In this study, we reprogrammed fibroblasts from an *RP2* patient carrying the nonsense mutation c.519C>T (p.R120X) into induced pluripotent stem cells (iPSC), and differentiated these cells into retinal pigment epithelial cells (RPE) to study the mechanisms of disease and test potential therapies. RP2 protein was undetectable in the RP2 R120X patient cells, suggesting a disease mechanism caused by complete lack of RP2 protein. The RP2 patient fibroblasts and iPSC-derived RPE cells showed phenotypic defects in IFT20 localization, Golgi cohesion and Gβ1 trafficking. These phenotypes were corrected by over-expressing GFP-tagged RP2. Using the translational read-through inducing drugs (TRIDs) G418 and PTC124 (Ataluren), we were able to restore up to 20% of endogenous, full-length RP2 protein in R120X cells. This level of restored RP2 was sufficient to reverse the cellular phenotypic defects observed in both the R120X patient fibroblasts and iPSC-RPE cells. This is the first proof-of-concept study to demonstrate successful read-through and restoration of RP2 function for the R120X nonsense mutation. The ability of the restored RP2 protein level to reverse the observed cellular phenotypes in cells lacking RP2 indicates that translational read-through could be clinically beneficial for patients.

## INTRODUCTION

Retinitis pigmentosa (RP) defines a clinically and genetically diverse group of inherited retinal dystrophies, which are characterized by progressive loss of visual function due to photoreceptor cell dysfunction and degeneration. The X-linked forms of RP (XLRP) are particularly severe, typically presenting in affected males in the first or second decade, with a rapid course of vision loss (1). Mutations in the XLRP gene *RP2* account for ∼15% of XLRP cases (2–9). Some RP2 patients have an early macular involvement, with early-onset macular atrophy and poor visual acuity in childhood (10,11).

The RP2 protein is ubiquitously expressed in human tissues and does not appear to be enriched in retina (12). However, patients with *RP2* mutations only have a retinal dysfunction, without any other apparent organ involvement, so an important question is why loss of RP2 leads specifically to RP. It is also unclear whether mutations in the ubiquitously expressed RP2 protein concomitantly affect photoreceptor and retinal pigment epithelium (RPE) cell function, or if one precedes the other, as it is expressed in both cell types (13,14). Interestingly, the clinical presentation of some *RP2* patients can resemble choroideremia (11), which is caused by the lack of Rab escort protein (REP1) and affects both the RPE and photoreceptors (15). Like REP1, RP2 has been implicated in vesicle traffic, potentially in cilia-associated traffic. A pool of RP2 is localized at the ciliary apparatus, basal body and cilium-associated centriole of photoreceptor cells (16–18) and RP2 also localizes to the Golgi, periciliary ridge and plasma membrane, implying a role for RP2 in primary cilia protein trafficking (16,17,19,20). RP2 acts as a GTPase activating protein (GAP) for the small GTPase Arl3 (21), which is essential for cilia function (13,22,23), further supporting the involvement of RP2 in cilia function.

The development of human induced pluripotent stem cell (iPSC) technology has greatly enhanced the potential for understanding disease mechanisms through the generation of specific cell types affected by a particular disease directly from patient cells. RPE cells are essential for visual function, with an important role in many processes; for example, the visual cycle and phagocytosis of outer segments (24–26). The dysfunction or death of RPE cells can cause a range of human retinal degenerative diseases, including retinitis pigmentosa. Therefore, human iPSCs differentiated into RPE cells present an opportunity to study retinal disease aetiology (27–30). The ability to derive specific cell types also facilitates the testing of promising drugs *in vitro* within the cellular and mutational context of the disease. This is particularly important for potential therapies that are sequence or mutation specific and would otherwise require the development of humanized, knock-in animal models.

No treatments are currently available to restore RP2 function in patients. RP2 patients frequently present with nonsense mutations (nine reported), and of these Arg120stop (R120X) is the most common, potentially as a mutation hotspot (3,31–35). Therapies that aim to restore translational read-through (TR) could therefore benefit a large group of RP2 patients. Nonsense mutations lead to premature termination codons (PTCs) that promote mRNA degradation through nonsense-mediated decay (NMD). NMD is a quality control pathway that targets transcripts in which translation is arrested, degrading potentially harmful mRNAs that have the potential to produce truncated polypeptides (36,37). Several compounds can suppress PTCs by inhibiting translationally active ribosomes, thereby hampering their proof-reading function, compromising the integrity of codon–anticodon pairing, which leads to the insertion of a near cognate tRNA codon at the stop codon (38). This results in the incorporation of an amino acid at the site of the mutation induced stop codon, and therefore, potentially, expression of a full-length protein (39,40). Aminoglycoside antibiotics, such as gentamicin (G418), have been shown to cause translational read-through of PTCs and restore full-length protein expression in a variety of contexts (41–44). Another promising translational read-through inducing drug (TRID) is the compound PTC124 or Ataluren (3-[5-(2-fluoro-phenyl)-[1,2,4]oxadiazole-3-yl]-benzoic acid). PTC124 was effective in restoring PTC read-through in several *in vitro* and *in vivo* studies for different diseases, including Duchenne muscular dystrophy and Usher syndrome (41,45–49). However, the feasibility of TRIDs needs to be evaluated for each respective disease and mutation, as the therapeutic outcome is influenced by the type of PTC, the genomic context of the nonsense mutation and the pathophysiology of the disorder.

Previously, we investigated the ability of aminoglycoside antibiotics to stimulate TR in RP2 R120X patient lymphoblast cells, but these drugs did not restore detectable levels of full-length RP2 protein (50). Recent advances in TRID technology, coupled to the ability to reprogramme patient cells into retinal cells, such as RPE that might be affected by the loss of RP2, and a greater understanding of RP2 function argue that it is timely to revisit the potential of TRIDs for RP2 stop mutations. Here, we characterized the phenotype of RP2 patient fibroblast and iPSC-derived RPE cells and tested for potential read-through of the R120X mutation and restoration of RP2 function.

## RESULTS

### Patient R120X fibroblasts are RP2-null cells with cilia-associated trafficking defects

**Figure 1.**
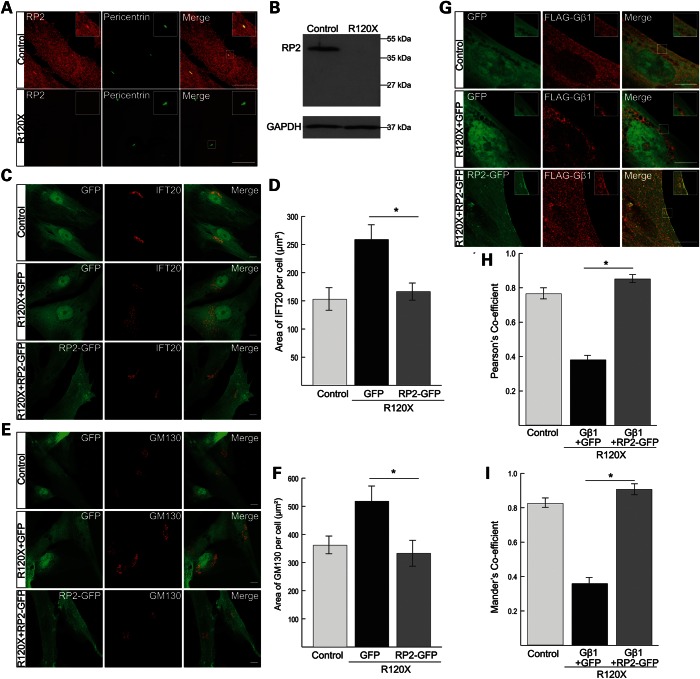
Characterization of RP2 R120X fibroblast cells. (**A**) RP2 (red) localizes to the plasma membrane and the basal body (pericentrin, green) in control fibroblasts, while R120X fibroblasts were negative for expression of RP2 by ICC. The inset shows a higher magnification of the basal body region boxed in the merge image. Scale bar 5 μm. (**B**) Western blot showing no RP2 protein was detected in R120X fibroblasts compared with control fibroblasts. GAPDH was used as a loading control (**C**) R120X or control cells were transfected by electroporation with empty GFP plasmid or RP2-GFP. R120X fibroblasts show dispersed IFT20 staining (red) compared with control cells when transfected with GFP alone. This phenotype was rescued by overexpression of RP2-GFP in R120X cells. Scale bar 10 μm. (**D**) IFT20 dispersal from the basal body was quantified by measuring the area of IFT20 per cell using a Threshold Plugin in ImageJ. (**E**) Cells were transfected as described above. Control and R120X fibroblasts were stained with an antibody for the *cis* Golgi component GM130 (red) to determine whether the loss of RP2 affects Golgi cohesion. GM130 shows a dispersed localization pattern in R120X fibroblasts compared with controls. This phenotype was rescued by overexpression of RP2-GFP. Scale bar 10 μm. (**F**) Golgi dispersal was analyzed as described above. (**G**) R120X or control fibroblast cells were transfected with FLAG-Gβ1 (red), GFP alone or RP2-GFP and FLAG-Gβ1. In R120X cells transfected with FLAG-Gβ1 and RP2-GFP, FLAG-Gβ1 localized to the plasma membrane, comparable to control cells. Scale bar 10 μm. (**H** and **I**) FLAG-Gβ1 plasma membrane staining was quantified by calculating the Pearson's and Mander's co-localization efficient in cells co-stained for FLAG and the plasma membrane marker cadherin, using the JaCOP plug-in and ImageJ software. *P ≤ 0.05, values are mean ± 2 SEM, *N* = 3 biological replicates.

Patient R120X fibroblasts were derived from a skin biopsy of a 24-year-old male. The nonsense mutation c.519C>T (p.R120X) was confirmed in the fibroblasts by direct sequencing (Supplementary Material, Fig. S1A). Fibroblast identity was confirmed by immunocytochemistry (ICC) using a fibroblast specific surface protein antibody (Supplementary Material, Fig. S1B). RP2 R120X patient fibroblasts and male control fibroblasts were analyzed for RP2 expression by ICC and western blotting. No RP2 staining was detectable by ICC in R120X fibroblasts compared with controls, which showed RP2 at the plasma membrane and basal body as previously described (16) (Fig. 1A). Furthermore, no full-length or truncated RP2 protein expression was detectable by western blotting (Fig. 1B). The RP2 antibody (S974) used in this study has been extensively characterized (12) and shown to detect RP2 protein fragments containing just the first 15 amino acids of RP2 (51). Knock-down of RP2 in ARPE19 cells by siRNA does not affect cilia incidence or cilia length (16). Similarly, no differences in cilia incidence or length were detected in R120X fibroblasts compared with controls (Supplementary Material, Fig. S1C–F). Earlier reports have suggested that knock-down of RP2 results in accumulation of the RP2 interacting protein Polycystin 2 (PC2) at the ciliary tip (17), and reduces the ciliary localization of Nephronophthisis 3 (NPHP3) (20). Therefore, we investigated whether localization of these proteins is affected in R120X fibroblasts. Staining for endogenous PC2 or NPHP3 revealed no qualitative or quantitative differences in staining pattern between ciliated R120X and control fibroblasts (Supplementary Material, Fig. S1G–J). Other reports showed that RP2 siRNA knock-down resulted in reduced Golgi cohesion and transducin subunit Gβ1 membrane association, as well as increased IFT20 dispersal from the basal body (16,52). Importantly, the R120X patient cells also showed mislocalization of IFT20 and Gβ1 and a disrupted Golgi morphology, as determined by quantitation of their area by unbiased ImageJ measurement of threshold staining (Fig. 1C–I). To determine whether these phenotypic defects were specific to the loss of RP2 function in the R120X patient fibroblasts, we performed a rescue experiment in which control and R120X fibroblasts were transfected with either RP2-GFP or empty GFP plasmid with subsequent staining for either IFT20 or the Golgi marker GM130. The localization of IFT20 at the basal body and Golgi morphology was rescued by overexpression of RP2-GFP in R120X fibroblasts, as assessed by a decrease in area compared with control levels (Fig. 1C–F). To investigate Gβ1 traffic, R120X patient cells were cotransfected with FLAG-Gβ1 with either RP2-GFP or empty GFP. In the absence of RP2, Gβ1 overexpression resulted in a mainly punctate, cytoplasmic staining pattern, as reported previously (52) (Fig. 1G); however, when Gβ1 was co-expressed with RP2-GFP, Gβ1 staining was observed at the plasma membrane and the level of co-localization with the plasma membrane marker pan-cadherin was increased, when measured by Pearson's correlation and the Mander's co-localization coefficient (Fig. 1G–I). These data confirm that these cellular phenotypes are a consequence of the lack of RP2 function in R120X patient-derived cells.

### Reprogramming RP2 R120X fibroblasts into iPSCs and differentiation into RPE cells

**Figure 2.**
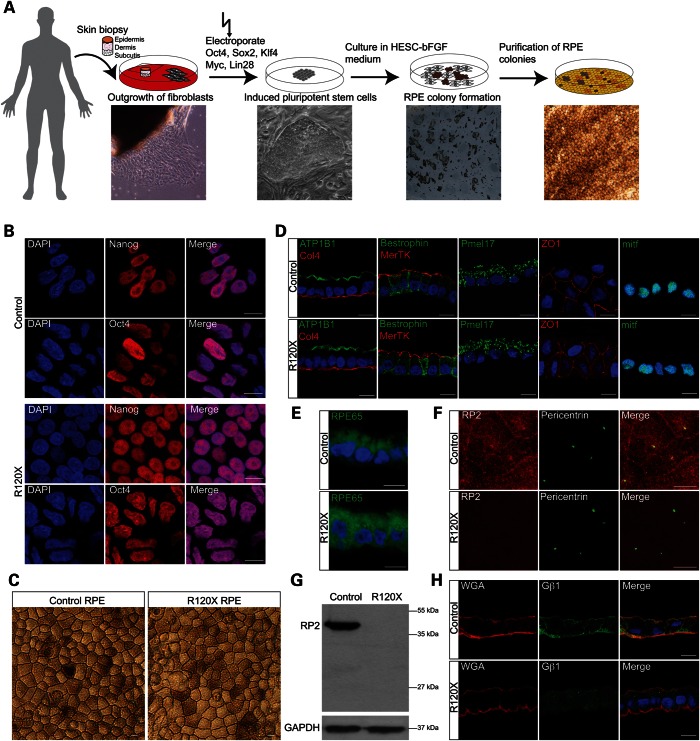
Generation of induced pluripotent stem cells (iPSCs) and retinal pigment epithelium cells (RPE) from RP2 R120X fibroblasts. (**A**) Schematic representation of iPSC and RPE cell generation from fibroblasts. A skin biopsy was taken and cut into small pieces using scalpel blades. The cells were then cultured in DMEM and outgrowing fibroblasts from the skin biopsy were isolated manually. To reprogram fibroblasts into iPSCs, cells were electroporated with plasmids containing Oct4, Sox2, Myc, Lin28 and Klf4, plated onto matrigel-coated dishes and cultured in mTESR medium. Forming iPSC colonies were isolated manually. iPSC were differentiated into RPE cells by culturing in HESC—bFGF medium. Pigmented RPE colonies were excised manually using crescent blades. (**B**) iPSC derived RPE from controls and the RP2 R120X patient showed nuclear (DAPI, blue) localization of the iPSC markers Nanog (red) and Oct4 (red). Scale bar 10 μm. (**C**) Differentiated control and R120X iPSC-derived RPE cells display the classic cobblestone pigmented morphology. Scale bar 10 μm. (**D**) Sectioned RPE cells were stained for RPE and differentiation markers: ATP1B1 (green), Col4 (red), Bestrophin (green), MerTK (red), Pmel17 (green), ZO1 (red), mitf (green) or (**E**) RPE65 (green). Scale bar 10 μm (**F**) RP2 protein is undetectable in iPSC-derived R120X RPE cells at the plasma membrane and the basal body by ICC. Scale bar 10 μm. (**G**) RP2 protein is undetectable in the iPSC-derived R120X RPE cells by western blotting. GAPDH was used as a loading control. (**H**) Sectioned RPE cells were stained for endogenous Gβ1 (green) the membrane marker wheat-germ agglutinin (WGA, red) and DAPI (blue). Gβ1 localizes to the plasma membrane in control RPE cells, but shows a diffuse cytoplasmic staining pattern in R120X RPE cells. Scale bar 10 μm.

To investigate the potential consequences of the loss of functional RP2 on retinal cells we reprogrammed patient fibroblasts into iPSCs and subsequently differentiated these into ciliated RPE cells (Fig. 2A). Briefly, R120X fibroblasts and control male fibroblasts were reprogrammed by electroporation with episomal plasmids containing Oct4, Sox2, Klf4, Myc and Lin28 (Fig. 2A). iPSC colonies were isolated and expanded. The pluripotency of the isolated iPSC lines was confirmed using iPSC-specific antibodies (Fig. 2B). RT-PCR confirmed induction of endogenous reprogramming genes and loss of episomal transcripts (Supplementary Material, Fig. S2).

Two clonal iPSC lines were differentiated into RPE cells by culturing the iPSC in HESC-bFGF medium. After ∼6 weeks, pigmented RPE colonies formed and were purified by manual excision using crescent blades and cultured until fully pigmented before further analysis (Fig. 2A). The isolated patient and control RPE cells displayed classic RPE cobblestone morphology and were pigmented (Fig. 2C). In addition, RPE cell morphology was confirmed using cell differentiation and RPE specific markers, such as Bestrophin, MerTK, the tight junction marker ZO-1, the melanosome marker Pmel17 (Fig. 2D) and the retinal isomerase RPE65 (Fig. 2E). No qualitative differences could be observed between the control and RP2 iPSC-derived RPE using these RPE markers. In addition, cilia incidence and morphology was comparable in both R120X and control RPE lines (Supplementary Material, Fig. S3A). In contrast, the R120X RPE cells had no detectable RP2 protein (Fig. 2G) and RP2 was absent from the basal body and the plasma membrane in these cells, compared with controls (Fig. 2F).

IFT20 localization and Golgi cohesion were compared in two independent iPSC-derived R120X RPE lines (Line 1 and Line 2) and two iPSC-derived RPE male controls (BJ and LPP). The area of pericentriolar IFT20 staining was increased in both R120X lines, with no significant difference between the two controls (Supplementary Material, Fig. S3B). Similarly, the area of three different Golgi markers (GM130, Giantin and TGN) was increased compared with controls in both R120X lines, showing disrupted Golgi cohesion (Supplementary Material, Fig. S3C–E). Staining for endogenous Gβ1 in control and R120X RPE cells showed that Gβ1 localized to the plasma membrane in control RPE cells, but had a diffuse cytoplasmic staining pattern in R120X RPE cells (Fig. 2H). These data also confirm that the R120X fibroblasts and RPE display similar cellular phenotypes that are specific and consistent.

### G418 and PTC124 can restore RP2 protein in patient R120X fibroblasts

**Figure 3.**
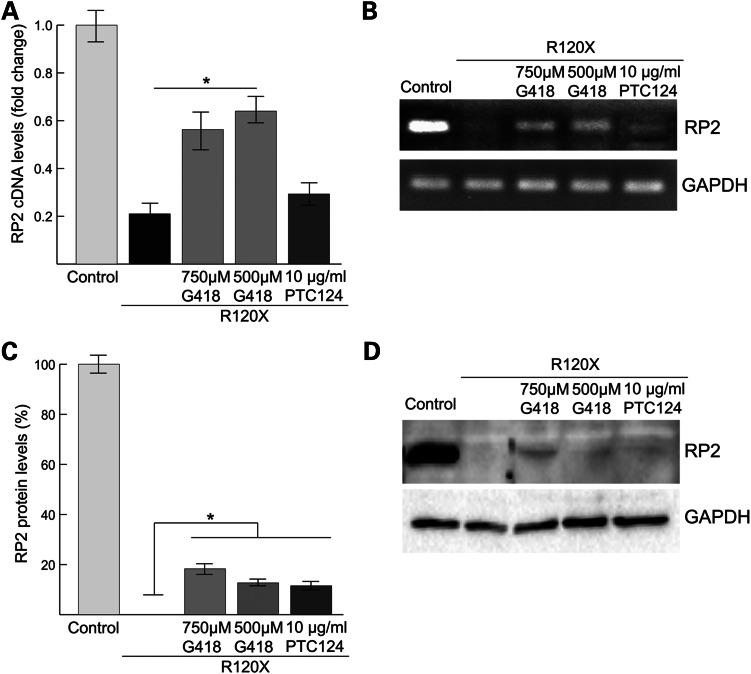
Endogenous full-length RP2 cDNA and protein can be restored in R120X fibroblasts by treatment with G418 and PTC124. (**A**) Treatment of R120X fibroblasts with a single 24 h dose of 500 μM G418 significantly increased RP2 mRNA levels compared with untreated cells. *P ≤ 0.05, values are mean ± 2 SEM, *N* = 3. (**B**) Agarose gel showing products of RT-qPCR amplification. GAPDH was used as an amplification control. (**C**) Treatment of R120X fibroblasts with a single 24 h dose of 750 μM, 500 μM G418 or 10 μg/ml PTC124 significantly increased RP2 protein levels compared with untreated R120X cells. *P ≤ 0.05, values are mean ± 2 SEM, *N* = 2. (**D**) Western blot showing increased RP2 protein levels in G418 and PTC124 treated R120X fibroblasts compared with untreated R120X cells.

Since no RP2 protein was detectable in the R120X cells by western blotting (Figs 1B and 2E), we reasoned that TRIDs, such as G418 and PTC124, could potentially restore some RP2 expression. Reverse transcriptase-quantitative PCR (RT-qPCR) showed that RP2 mRNA levels in R120X fibroblasts were ∼80% lower than levels in male control cells, suggesting that the mutant RP2 R120X transcript is subjected to NMD (Fig. 3A and B). A dose response over 24 h revealed that the most effective dose of 750 or 500 μM G418 resulted in an ∼40% increase in R120X RP2 mRNA levels (Fig. 3A and B). In contrast, the most effective dose of PTC124 treatment (10 μg/ml) appeared to increase RP2 mRNA levels compared with untreated R120X cells; however, this did not reach statistical significance (Fig. 3A and B). These data, at the most effective doses tested for the compounds, suggest a difference in potency or different mechanism of action for the TRIDs G418 and PTC124. Next, we analyzed RP2 protein expression in R120X fibroblast cells by western blotting following 24-hour treatment with G418 or PTC124. The amount of restored full-length RP2 protein was quantified using a calibration curve derived from purified recombinant HIS-tagged RP2 (Supplementary Material, Fig. S4A). Treatment with G418 (750 μM dose) or PTC124 (10 μg/ml dose) led to an increase in RP2 protein to 20% and 13% of control cell levels, respectively (Fig. 3C and D).

### TRID mediated correction of the RP2-null cellular phenotype

**Figure 4.**
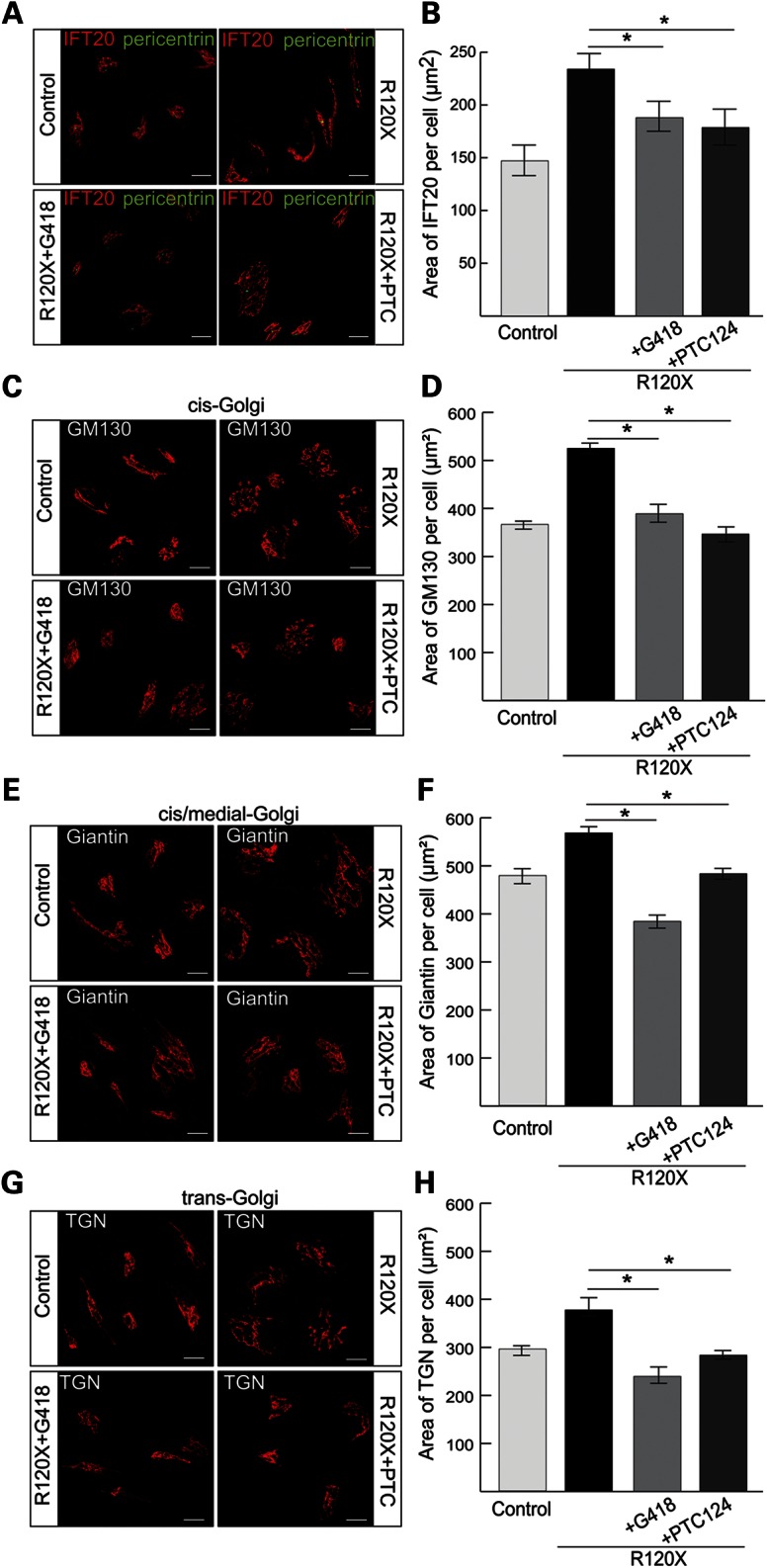
Cellular effects of G418 or PTC124 on R120X fibroblast cells. (**A**) R120X fibroblasts were treated with a single 24 h dose of either 750 μM G418 or 10 μg/ml PTC124 and stained for IFT20 (red) and pericentrin (green). Scale bar 10 μm. IFT20 is dispersed in untreated R120X fibroblasts compared with control cells. This phenotype was reversed by treatment with G418 or PTC124. (**B**) Quantitation of the area of IFT20 staining showing significant improvement of IFT20 dispersal following treatment with either drug. *P ≤ 0.05, values are mean ± 2 SEM, *N* = 3 independent experiments with 150 cells per experiment. (**C**, **E** and **G**) Control and R120X fibroblasts were stained with antibodies for the *cis* (C; GM130), *cis*-medial (E; Giantin) and *trans* Golgi (G; TGN) components (red) to determine disruption to any part of the Golgi. Scale bar 10 μm. (**D**, **F** and **H**) Quantitation of Golgi area staining showing that in untreated R120X cells the area of all three Golgi components per cell is significantly increased compared with control fibroblasts, indicating lack of Golgi cohesion in these RP2-null cells. Treatment with either G418 or PTC124 restored the R120X Golgi area to control levels. *P ≤ 0.05, values are mean ± 2 SEM, *N* = 3 independent experiments with 150 cells per experiment.

In order to determine if the increased RP2 transcript and protein levels following treatment with G418 and PTC124 resulted in a rescue of functional RP2, and were sufficient to reverse the phenotypic effects, we investigated the cellular phenotype in TRID treated R120X fibroblasts compared with untreated cells. Initially, to test if G418 or PTC124 had any off target effects on these phenotypes, we treated control fibroblasts with a single 24 h dose of either 750 μM G418 or 10 μg/ml PTC124, and IFT20 localization at the basal body and Golgi cohesion were examined. Neither of the TRID treatments had any effect on these cellular markers in control male fibroblast cells (Supplementary Material, Fig. S4B–E). As before, untreated R120X fibroblasts showed a significantly larger IFT20 pericentriolar area, compared with controls (Fig. 4A). Following TRID treatment the average area of IFT20 in each R120X fibroblast cell was significantly reduced and comparable to control fibroblasts (Fig. 4A and B). Similarly, Golgi area, assessed by three Golgi markers, was reduced following treatment of R120X fibroblasts with either G418 or PTC124 (Fig. 4C–H).

**Figure 5.**
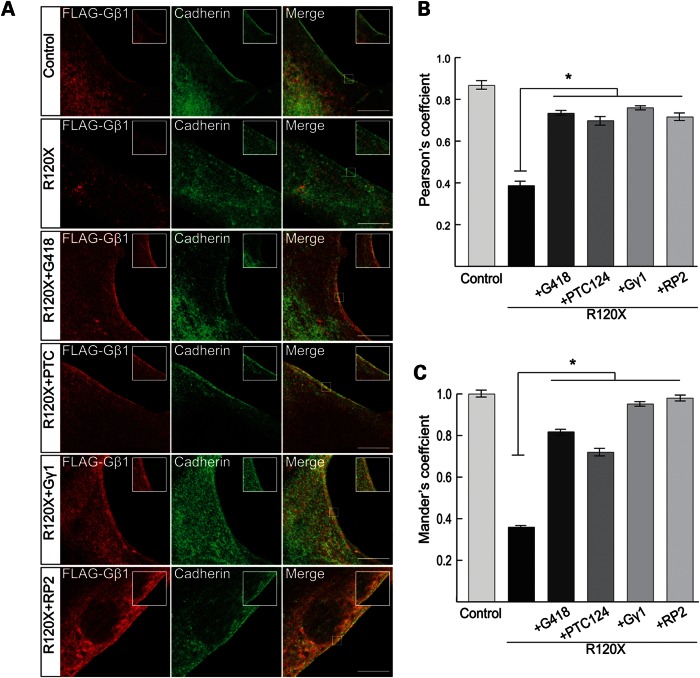
G418 and PTC124 restore sufficient RP2 function to facilitate plasma membrane association of Gβ1. (**A**) Immunofluorescence showing FLAG-Gβ1 association with the plasma membrane when expressed in control fibroblasts, or when co-expressed with RP2 or HA-Gγ1 in R120X fibroblasts. FLAG-Gβ1 was detected with FLAG antibody (red) and the plasma membrane is highlighted with anti-cadherin (green). Treatment with a single 24 h dose of either 750 μM G418 or 10 μg/ml PTC124 restored FLAG-Gβ1 plasma membrane localization in the absence of RP2 or HA-Gγ1. The inset shows a higher magnification of the region boxed in the merge image. Scale bar 10 μm. (**B** and **C**) Quantitation of Gβ1 plasma membrane association calculated using Pearson's (B) and Mander's (C) co-localization coefficients for FLAG and pan-cadherin, using the JaCOP plug-in and ImageJ software. *P ≤ 0.05, values are means ± 2 SEM, *N* = 3 biological replicates.

To investigate the effect of TRID mediated RP2 rescue on Gβ1 trafficking, R120X fibroblasts were transfected with FLAG-Gβ1 and treated with a single 24 h dose of G418 (750 μM) or PTC124 (10 μg/ml). Cells were then stained for FLAG and pan-cadherin, which was used as an independent plasma membrane marker (Fig. 5A). Untagged RP2 and HA-Gγ1 were used as positive controls, since expression of Gβ1 with HA-Gγ1 results in the translocation of Gβ1 from the cytosol to the plasma membrane, irrespective of whether RP2 is present (52). Transfection of R120X fibroblasts with FLAG-Gβ1 alone showed mainly cytosolic Gβ1 staining, whereas FLAG-Gβ1 localized to the plasma membrane in control cells and co-localized with pan-cadherin, as measured with Pearson's and Mander's coefficients, in the presence HA-Gγ1 or RP2 (Fig. 5A). Treatment of FLAG-Gβ1 transfected R120X fibroblasts with G418 or PTC124 also restored plasma membrane association of FLAG-Gβ1 in R120X cells (Fig. 5A). Co-localization of FLAG-Gβ1 and cadherin was quantified and confirmed that treatment with either G418 or PTC124 restored FLAG-Gβ1 plasma membrane localization in R120X cells to levels similar to those in control cells (Fig. 5B and C). These findings further confirm that the mislocalization of Gβ1 in R120X fibroblasts is due to lack of RP2 and that overexpression of RP2 or HA-Gγ1 can enhance the traffic of Gβ1 to membranes. In addition, both translational read-through drugs can restore sufficient endogenous RP2 expression and function to facilitate the trafficking and plasma membrane association of Gβ1 in RP2-null R120X cells.

### G418 and PTC124 restore RP2 function in R120X iPSC-derived RPE cells

**Figure 6.**
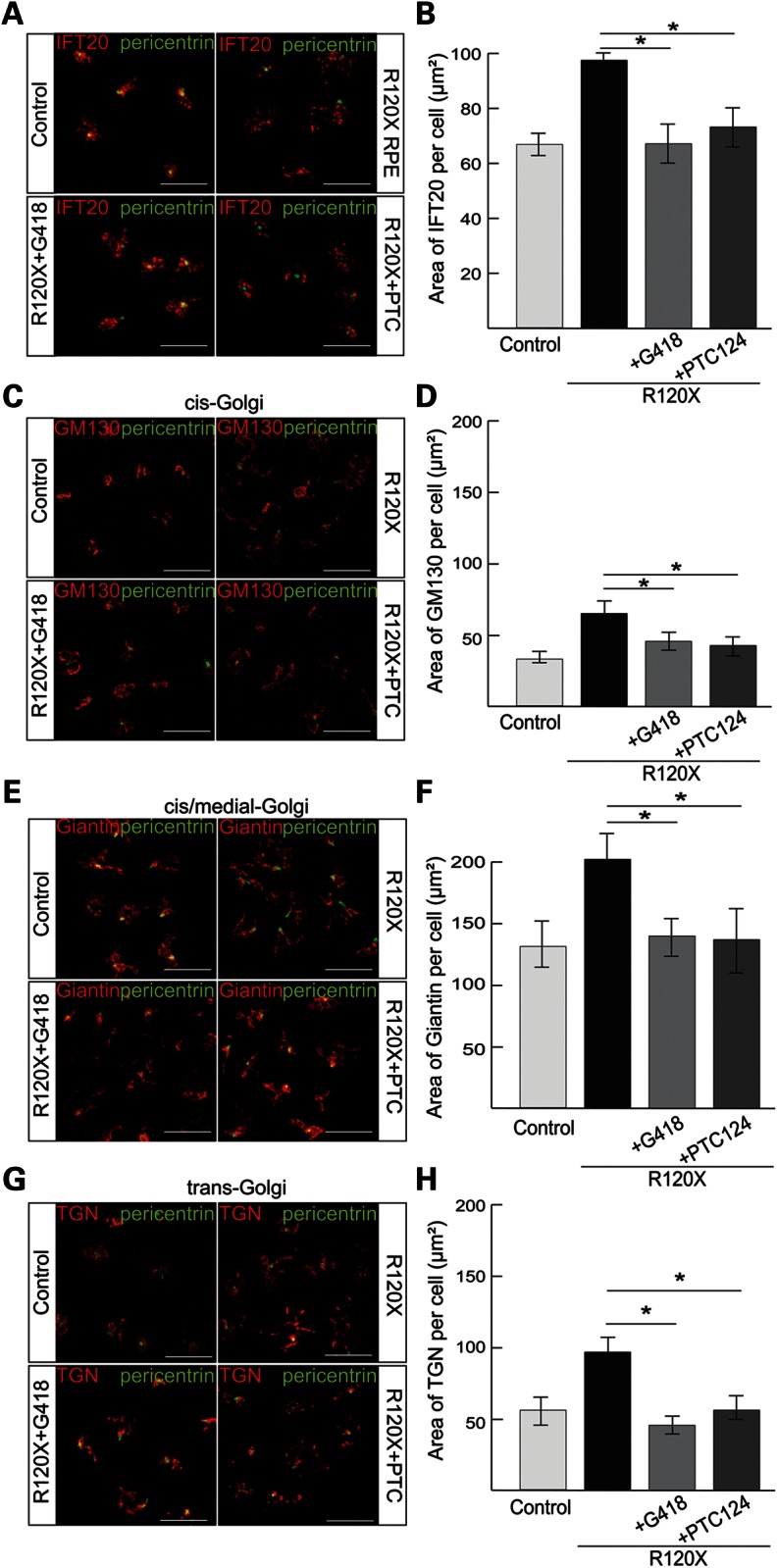
Suppression of IFT20 and Golgi defects with TRIDs in R120X RPE cells. (**A**) R120X iPSC-RPE were treated with a single 24 h dose of either 750 μM G418 or 10 μg/ml PTC124 and stained for IFT20 (red) and pericentrin (green). Scale bar 10 μm. (**B**) IFT20 area was quantified using ImageJ. IFT20 is significantly dispersed in untreated R120X RPE compared with control RPE cells. This RP2-null cellular phenotype is reversed by treatment with either G418 or PTC124. *P ≤ 0.05, values are means ± 2 SEM, *N* = 3 independent experiments with 300 cells per experiment. (**C**, **E** and **G**) Control and R120X RPE cells were stained (red) with antibodies for the *cis*, *cis*-medial and *trans* Golgi components (C; GM130, E; Giantin and G; TGN) and pericentrin (green) to assess disruption of any part of the Golgi. Scale bar 10 μm. (**D**, **F** and **H**) Golgi area was quantified using ImageJ. In untreated R120X RPE cells the area of all three Golgi components per cell is significantly increased compared with control RPE cells, indicating lack of Golgi cohesion in these cells. Treatment with G418 or PTC124 restored Golgi cohesion to levels comparable to control RPE cells. *P ≤ 0.05, values are means ± 2 SEM, *N* = 3 independent experiments with 300 cells per experiment.

**Figure 7.**
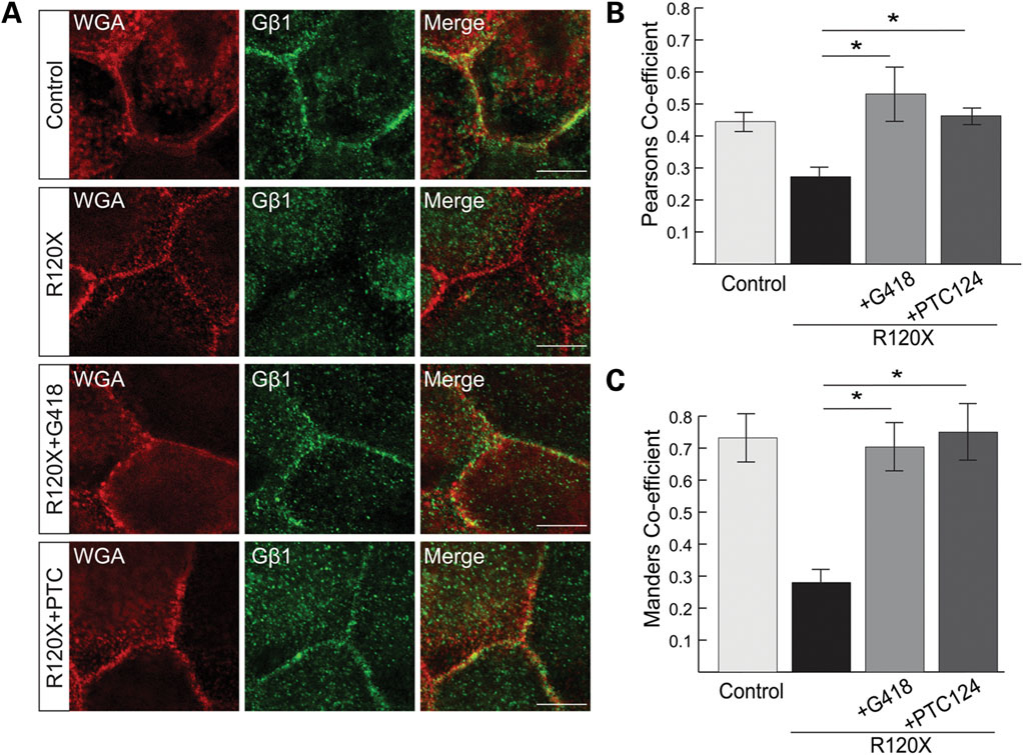
TRID treatment can enhance endogenous Gβ1 traffic in R120X iPSC-RPE. (**A**) Control and R120X RPE cells were stained for endogenous Gβ1 (green) and the membrane marker WGA (red). Gβ1 plasma membrane localization is disrupted in R120X RPE cells, but restored with treatment of either G418 or PTC124. (**B** and **C**) Gβ1 plasma membrane staining was quantified by calculating the Pearson's (B) and Mander's (C) co-localization coefficient in cells co-stained for Gβ1 and the plasma membrane marker WGA, using the JaCOP plug-in and ImageJ software. *P ≤ 0.05, values are mean ± 2 SEM, *N* = 3 independent experiments with 300 cells per experiment.

The effect of the TRIDs on the RP2-null R120X iPSC-RPE cellular phenotype was investigated. A single 24 h dose of either G418 (750 μM) or PTC124 (10 μg/ml) was able to restore tight IFT20 localization at the basal body and to reduce dispersal of Golgi components, thereby rescuing the RP2-null phenotype in patient-derived R120X RPE cells to that of wild-type iPSC-derived RPE cells (Fig. 6A–H). In addition, plasma membrane localization of endogenous Gβ1 in R120X RPE cells was restored following treatment with G418 and PTC124 (Fig. 7A), and co-localization with the plasma membrane marker wheat-germ agglutinin (WGA) was confirmed by both Pearson's and Mander's coefficients (Fig. 7B and C). Both TRIDs showed the same efficiency in these functional assays at the doses tested, indicating that translational read-through therapy could be clinically beneficial for patients.

## DISCUSSION

In this study we used patient cells to investigate the cellular defects associated with an RP2 R120X mutation that causes XLRP, and tested a potential therapy. We characterized fibroblast cells and iPSC-derived RPE cells and confirmed that they are RP2-null cells with no functional RP2 protein. The disease mechanisms resulting from the loss of RP2 are still unclear. Although it is widely assumed that the rod photoreceptors are the site of primary pathology, the effect of RP2 loss on cone photoreceptor and RPE function cannot be disregarded, especially given the prevalence of macular atrophy and choroideremia-like symptoms associated with loss of RP2 function (11). Several retinal degenerative diseases are caused by malfunction of the photoreceptors, RPE or both. For example, choroideremia is an X-linked retinal dystrophy, which is characterized by progressive degeneration of the choriocapillaris, retinal pigment epithelium and photoreceptors (15). Recently, a mouse knock-out model for RP2 was developed and reported to have cone cell dysfunction and degeneration with cone opsin mislocalization (53); however, the progression of disease in this model appears to be slower than that observed in XLRP patients. Therefore, detailed investigation of patient cells could reveal the basis for this difference and enable us to study distinct retinal cell types in isolation.

Similar to siRNA mediated RP2 knock-down in ARPE19 cells (16), both the patient R120X fibroblasts and iPSC-derived RPE cells had a less compact Golgi and IFT20 was dispersed from around the basal body. These phenotypes could be rescued by overexpression of RP2, indicating that they are a consequence of the loss of RP2 protein. In ciliated cells, RP2 localizes to the periciliary ridge, the basal body and the Golgi complex (16,17,20). These structures function in association with each other to control protein traffic in ciliated cells (54,55), which suggests a role for RP2 in pericentriolar vesicle trafficking and intraflagellar transport (IFT). The molecular basis for the alteration in Golgi morphology, however, is not currently understood. RP2 is a GAP for Arl3, and expression of a constitutively active form of Arl3 (Arl3-Q71L) also results in disruption of Golgi cohesion and dispersal of IFT20 from the basal body (16). Therefore, it is likely that dysregulation of the GTPase activity of Arl3 underlies these changes in Golgi morphology and IFT20 localization.

The incidence and length of cilia were not affected by the loss of RP2. Therefore, RP2 might be specifically involved in the traffic of a subset of cilia-associated proteins. These candidate proteins include; the visual G protein transducin beta subunit, Gβ1, (52), PC2 (17) and NPHP3 (20). PC2 and NPHP3 localization was unaffected by the loss of RP2. In contrast, RP2-null patient cells had problems efficiently trafficking Gβ1 to the plasma membrane. RP2 can bind Gβ1 and Gβ1 is released from RP2 by Arl3-Q71L. Arl3 and its effectors Unc119 and PDE6δ are involved in the membrane traffic of lipidated proteins, including the alpha (acyl) and gamma (prenyl) subunits of transducin (56,57). Taken together with previous studies on G protein trafficking, we proposed a model for trafficking of membrane-associated proteins in photoreceptors in which RP2 acts with Arl3, Unc119 and PDE6δ, to target the assembly and traffic of membrane-associated cargo proteins, such as heterotrimeric transducin, to specific membranes and co-ordinate protein traffic (58,59). No differences in G protein traffic were observed in the retina of RP2 knock-out mice (53), so it remains unclear if this discrepancy with the human cells reflects a difference in function between human and mouse RP2, or if it is related to the levels of G protein subunits in the experimental systems, as overexpression of Gγ1 can efficiently replace RP2 to facilitate Gβ1 membrane association. Here we show that endogenous Gβ1 trafficking to the plasma membrane of RPE cells is dependent on functional RP2 in human retinal cellular context.

A major advantage of patient-derived cells is that they enable testing of sequence, or mutation-type, dependent-specific therapies, such as read-through therapies, that could not be tested on knock-out animal models. Therefore, we analyzed the effects of RP2 loss and treatment with TRIDs (G418 and PTC124) on patient-derived fibroblasts and iPSC-derived RPE cells, which displayed the same morphological characteristics as typical RPE cells.

G418 is an aminoglycoside that is proposed to suppress NMD by inhibiting translationally active ribosomes and thereby lowering the efficiency of the cellular proof-reading machinery (60). G418 has successfully suppressed PTCs in cellular models (61), as well as in patients with Duchenne muscular dystrophy and cystic fibrosis (62–66). However, the clinical disadvantages of G418 treatment are the high level of toxicity for long-term use, such as nephron- and ototoxicity, as well as the requirement for intramuscular or intravenous drug delivery (67). In contrast, PTC124 has an excellent preliminary safety and tolerability profile and can be taken orally (47,68). PTC124 has been successfully used in treatment of arylsulfatase B (ARSB associated mucopolysaccharidosis) (69) and PTC124 is currently in clinical trials for the treatment of cystic fibrosis (70) and Duchenne muscular dystrophy (46); however, the clinical findings of these studies are not conclusive. In a Phase 3 clinical trial of PTC124 treatment for cystic fibrosis, patients did not have an increased lung function following treatment (70). In contrast, the majority of patients in a Phase 2a clinical trial for Duchenne muscular dystrophy showed a PTC124 mediated increase in dystrophin levels (46). The mechanism of action for PTC124 is still controversial. It has been suggested that the activity of PTC124 in luciferase-based *in vitro* experiments is due to PTC124′s post-translational stabilization of the luciferase reporter and not a genuine read-through effect (71). However, PTC124 treatment restored 20–25% of dystrophin protein levels in the mdx-mouse, a model for Duchenne muscular dystrophy, *in vitro* and *in vivo* (47). In addition, a recent study using a GFP reporter plasmid showed that PCT124 was effective in inducing full-length protein expression of GFP containing a stop codon. Computational modelling of the supramolecular interaction of PTC124 and an mRNA fragment containing a stop codon confirmed that PTC124 interacts specifically with the stop codon (72). These findings and our data suggest that PTC124 is indeed able to promote translational read-through of premature stop codons.

There is great potential for the topical application of TRIDs to the eye. In a model of human Usher syndrome type 1C (USH1C) treatment with PTC124 was able to rescue translational read-through of a patient mutation in the *USH1C* gene and restored the functional properties of the protein (48,73). Furthermore, in a recent study of a mouse model with aniridia (pax6 mouse) topical administration of PTC124 inhibited disease progression and even reversed ocular malformations (49).

Treatment of R120X fibroblasts with the established TRID, G418, caused an increase in RP2 mRNA (40%) and protein levels (20%) while treatment with PTC124 increased RP2 protein levels (13%), but did not significantly increase RP2 mRNA levels. One possible explanation for this could be a difference in drug potency or mechanism of action. It is possible that G418 increases the rate of translational read-through or stabilizes RP2 mRNA transcripts more efficiently than PTC124, reflecting the slightly higher amount of RP2 protein. Also PTC124 and G418 potentially act via different mechanisms in preventing NMD, for example, by inducing cleavage and inactivation of eukaryotic initiation factors or blocking ribosomal subunits involved in transcription. Interestingly, PTC124 is able to promote read-through of all three nonsense codons, with the highest read-through of stop codon UGA, which is the R120X stop codon, followed by UAG and then UAA (47).

In this study, we restored ∼13–20% of RP2 protein in R120X patient cells, similar to the level of restored dystrophin protein achieved by PTC124 treatment in the mdx mouse (47). Of the 13–20% of restored RP2 protein, only a fraction is likely to be the same sequence as wild-type protein, while the rest of the full-length RP2 could harbour different amino acids at position 120, since the suppression of stop codons occurs through the random mispairing of a near cognate tRNA with the stop codon. Therefore, probably much less than 13–20% of full-length, wild-type sequence RP2 protein were restored, but importantly our data show that only low levels of full-length functional RP2 are required to rescue the observed phenotypes.

Furthermore, we were able to demonstrate that the TRIDs restored full-length protein, was functional. This was possible through advances in understanding the function of RP2, which showed that loss of RP2 in cells causes IFT20 and Golgi dispersal, as well as mislocalization of Gβ1 (16,52). These findings provide a vital tool to assess the efficacy of TRIDs to restore functional RP2 protein and therefore reversal of the observed disease phenotypes. The development of iPSC patient cell lines will enable the investigation of other cellular consequences of RP2 loss, and in defining the molecular basis for disease. Further investigation of the RPE biology will determine if these cells are fully functional, for example, in photoreceptor phagocytosis, visual cycle function and ion homeostasis. It might also be possible to differentiate these iPS cells into photoreceptors and investigate their biology. Photoreceptors are highly specialized ciliated cells and their cilia are complex organelles, which require efficient protein trafficking. It is possible that RP2 is required to fulfil these specialized needs, and it will be important to test whether these TRIDs have a significant effect *in vivo* with further detailed investigation.

In conclusion, this proof-of-concept study has demonstrated that small molecules, such as PTC124, and aminoglycosides, such as G418, can promote read-through of PTC in *RP2* and can therefore potentially restore gene function in patients. This, together with the ability to differentiate RPE cells from patient-derived iPS cells provides a compelling case to evaluate the potential therapeutic value of this approach.

## MATERIALS AND METHODS

### Study subjects

After informed consent a skin biopsy was obtained from a male RP2 patient with a diagnostically confirmed R120X mutation, and from healthy control male individuals. The study followed the tenets of the Declaration of Helsinki and was approved by the Moorfields and Whittington Hospitals' local Research Ethics Committees and the NRES Committee London Riverside Ethics Committee (REC 12/LO/0489).

### Reagents

G418 was obtained from Sigma-Aldrich and cells were treated with a single 24 h dose. PTC124 (Ataluren) was obtained from Selleckchem (Boston, USA). Cells were treated with a single 24 h dose before analysis. LabTAQ High Green Rox (Labtech, Uckfield, UK) was used for qPCR amplifications.

### Cell culture of fibroblasts from skin biopsies

Five millimeter punch biopsies were obtained from an RP2 patient and a healthy male control under local anaesthetic. The biopsies were sectioned into smaller (∼1 mm) pieces using sterile scalpel blades and placed into a 6-well plate. The tissue was exposed to a minimal amount of fibroblast growth media (DMEM, 10% foetal calf serum, 1 mm non-essential amino acids, 1 mm Glutamax™ and penicillin–streptomycin, Life Technologies, Germany), overlaid with a sterile coverslip overnight and incubated at 37°C in 5% CO_2_ in a humidified incubator. The following day 2 ml of fresh medium was added. The tissue was cultured until sufficient fibroblast outgrowth had occurred (∼4 weeks) for further passage of cells. Fibroblast cells were washed in PBS, dissociated using TrypLE™ Express (Life Technologies, Germany) and re-plated at a split ratio of 1:3. For maintenance, the media was replaced every 3–4 days and cells passaged when 80–90% confluent. gDNA was extracted from fibroblast cells using the GeneEute™ Mammalian Genomic DNA miniprep Kit (Sigma) and used in direct sequencing to confirm the RP2 mutation. For immunofluorescence staining, cells were cultured in 8-well chamber slides (VWR, Lutterworth, UK). Cells for western blotting were plated into 6-well plates (Nunc, Fisher Scientific, UK). For rescue and Gβ1 trafficking experiments, fibroblasts were transfected by electroporation with 2 μg of Gβ1-FLAG and 2 μg of either GFP alone, RP2-GFP or Gγ1-HA.

### Reprogramming patient-derived skin cells to IPSCs

iPSCs were generated from fibroblast cells in growth phase using integration-free episomal vectors obtained from Addgene: pCXLE-hOCT3/4-shp53-F (Addgene plasmid 27077), pCXLE-hSK (Addgene plasmid 27078) and pCXLE-hUL (Addgene Plasmid 27080) (74). Fibroblast cells were dissociated in TrypLE™ Express, resuspended in PBS and counted using a haemocytometer. Of note,1 × 10^6^ cells were isolated, resuspended in 100 μl Nucleofector^®^ solution from the Cell Line Nucleofector^®^ Kit R (Lonza) and mixed with 1 μg of each episomal plasmid. Samples were transferred into a cuvette and electroporated using an Amaxa Nucleofector I device. Following electroporation cells were plated onto a 0.2% gelatin-coated 10 cm^2^ dish, cultured in fibroblast growth medium containing 0.5 mm sodium butyrate at 37°C within a 5% CO_2_ humidified incubator. The medium was changed daily. On Day 7 the cells were dissociated using TrypLE™ Express and plated onto 6-well plates coated with Corning^®^ Matrigel^®^ basement membrane matrix (Corning) at a density of 2 × 10^5^ cells per well and maintained overnight in fibroblast growth medium. The following day the media was changed to mTeSR1 (Stem Cell Technologies), which was replaced daily until iPSC colonies appeared (approximately at Day 30). Clonal iPSC colonies were identified by morphology and StainAlive DyLight™ TRA-1-81 staining (Stemgent) and isolated using mechanical dissection. iPSC lines were maintained initially in mTeSR1 using mechanical dissection for the first four passages, after which dispase enzymatic passaging was performed.

### Differentiation of iPSCs to RPE

iPSC were differentiated into RPE cells by spontaneous differentiation using an established protocol (28,30). RPE cells were purified by manual isolation of pigmented colonies using crescent blades (Fine Science Tools). Colonies were dissociated by incubation in Accutase solution (Sigma-Aldrich) for 2–3 h and plated at a density of 50 000 cells/cm^2^ in X-Vivo 10 onto Matrigel-coated tissue culture plastic dishes. The media was replaced twice weekly for ∼6–8 weeks, until cells had developed a confluent pigmented monolayer.

### RT-qPCR

To determine the level of RP2 transcript and the efficiency of the TRIDs treatment, total RNA was extracted from control and R120X (TRID treated and untreated) fibroblasts using RNeasy extraction kit (Qiagen, Crawley, UK) according to the manufacturer's instructions. cDNA was reverse transcribed using a cDNA synthesis kit (Bioline, London, UK) with a random hexamer primer mix. For quantitative real-time PCR amplifications RP2 was amplified rom exon 1 to exon 2 using the following, intron spanning primers: 5′-AAGCAGTACAGCTGGGATCA-3′ and 5′-TCTTGAATGAGAAACTGTTGTCC-3′. GAPDH and β-tubulin were used as amplification controls and to normalize RP2 expression using the following forward and reverse primers: GAPDH 5′-CCCCACCACACTGAATCTCC-3′ and 5′-GGTACTTTATTGATGGTACATGACAAG-3′; β-tubulin 5′-AATCCCCACCTTTTCTTACTCC-3′ and 5′-AAAGATGGAGGAGGGTTCCC-3′. Quantitative PCR reactions were run on a StepOne Plus Real Time PCR System (Life Technologies) and analyzed using the Comparative C_T_ experiment option in the StepOne software (Version 2.3). For the analysis of iPSC gene expression in clonal lines RNA was extracted from undifferentiated iPSC cultures and converted into cDNA using the SuperScript Reverse Transcriptase III Kit (Life Technologies) using oligo-dT priming according to the manufacturer’s instructions. Primers specific to the episomal reprogramming vector or endogenous coding sequence of Oct4, Klf4, Sox2, Lin28 and c-myc (74) and control genes (GAPDH, β-tubulin and B2M; (28) were used to amplify iPSC cDNA in the StepOne Plus Real Time PCR System using Power SYBR^®^ Green Master Mix (Life Technologies, Germany) according to the manufacturer's instructions. Pluripotency gene expression was normalized to the multiple control genes and endogenous expression plotted as the target expression relative to the episomal vector.

### Antibodies

Production and characterization of affinity-purified sheep polyclonal RP2 antisera S974 has been described previously (12,51). Mouse anti-M2 FLAG (1:1000, Sigma-Aldrich) was used to detect FLAG-Gβ1. The guinea-pig anti-IFT20 (1:200) antibody was a gift from Dr J. C. Besharse (Cell Biology Department, Medical College of Wisconsin, Milwaukee, USA). The following antibodies were used as Golgi markers: GM130 (1:100, Clone 35, BD Biosciences, Oxford, UK), TGN-46 (1:1000, Abcam), Giantin (1:500, Abcam). Rabbit pan-cadherin (1:500, Abcam) was used to stain membrane structures. Rabbit anti-pericentrin (1:2000, Abcam) and mouse anti-pericentrin (1:1000, Abcam) were used as centrosomal/basal body markers. The mouse anti-fibroblast surface protein antibody (1:100) was purchased from Sigma. The rabbit anti-Arl13b antibody (1:500, Protein TechGroup) and mouse-anti acetylated alpha tubulin (1:1000, Sigma-Aldrich) were used as cilia markers. Goat anti-Polycystin-2 (1:50) and rabbit anti-Gβ1 antibody (1:200) were purchased from Santa Cruz and rabbit anti-NPHP3 (1:100) was from Proteintech. Pluripotency markers for iPSC staining were: rabbit anti-Oct4 (1:1000, Abcam) rabbit anti-Nanog (1:1000, Abcam), mouse anti-Tra-1-60 (1:1000, Life Technologies), mouse anti-Tra-1-81 (1:1000, Life Technologies). Markers for staining of RPE cells were: mouse anti-mitf (1:300, Neomarkers, Fremont, CA), mouse anti-Pmel17 (1:1000, DAKO), rabbit anti-ZO1 (1:500, Invitrogen), mouse anti-Bestrophin (1:1000, Abcam), rabbit anti-MerTK (1:50, Abcam), mouse anti-ATP1B1 (1:100, Abcam), mouse anti-RPE65 (MAB5428, 1:500, Merck Millipore, Watford, UK).

### Western blotting

For western blotting of RP2 or GAPDH protein expression, crude sheep anti-RP2 antibody (1:2000) or mouse anti-GAPDH antibody (1:40 000, Sigma-Aldrich) were used respectively. Secondary antibodies used were horseradish peroxidase (HRP) conjugated goat anti-sheep, donkey anti-rabbit or goat anti-mouse antibodies (Stratech). Blots were developed using the enhanced chemiluminescence (ECL) western blotting detection system (GE Healthcare). Protein levels were quantified using ImageJ software.

### Immunocytochemistry (ICC)

For ICC, cells were washed twice in PBS and either fixed in 100% ice-cold methanol for 2 min or 4% paraformaldehyde (PFA) for 10 min. For sectioning, RPE cells were washed in PBS and fixed in the cell culture well in 4% PFA for 30 min at 4°C. The RPE monolayer was carefully lifted off with a cell scraper and washed gently in 30% sucrose. Fresh 30% sucrose was added and RPE cells stored at 4°C over night. The RPE monolayer was embedded in O.C.T. compound (VWR), frozen in a dry ice/acetone slurry stored at −80°C prior to cryosectioning at 10 μm onto Superfrost plus slides (VWR). RPE sections and other cells were permeabilized with 0.2% Triton X-100 in PBS for 10 min and subsequently incubated for 1 h in blocking buffer [3% bovine serum albumin (BSA), 10% normal donkey serum in PBS] to avoid non-specific antibody binding. Following block, cells were incubated for 1 h with primary antibodies at the appropriate titre. After washing with PBS cells were then incubated with fluorescent-labelled (Cyanine2 or Cyanine3) secondary antibodies (1:100, Stratech, Newmarket, Suffolk, UK) for 1 h in 3% BSA in PBS. Following several washes with PBS, cells were incubated for 5 min with 2 μg/ml 4′,6-diamidino-2- phenylindole (DAPI, Sigma-Aldrich) in PBS to stain the nuclei. Confocal images were obtained using the LSM700 microscope (Carl Zeiss MicroImaging) and analyzed using the LSM Image Browser software (Carl Zeiss MicroImaging), prior to export and image processing and annotation using Adobe Photoshop and Illustrator. Co-localization of proteins at the plasma membrane was determined by morphological assessment of fluorescently labelled cells using a Nikon Eclipse 80i microscope and Nikon NIS-Elements software (Basic Research, Version 2.2, Nikon). The Pearson's correlation coefficient and the Mander's co-localization coefficient were determined to corroborate the morphological assessment and quantify co-localization, as previously described (75). Briefly, per treatment a minimum of three biological replicates were analyzed using the ImageJ JACob plugin software. The area of IFT20 and Golgi staining was analyzed for each organelle using a confocal projection and the Threshold plugin in ImageJ.

### Statistical analysis

Statistical analyses were performed using the excel software. Quantitative PCR reactions were analyzed using the Comparative C_T_ experiment option in the StepOne software (Version 2.3). Significance was determined by Student's *t* test and co-localization correlations were determined using Pearson's and Mander's correlations in ImageJ with the JACoP plug-in. Data are presented as means ± 2 SEM. Significance levels were set when *P* < 0.05 (*), *P* < 0.01 (**), *P* < 0.001 (***).

## SUPPLEMENTARY MATERIAL

Supplementary Material is available at *HMG* online.

## FUNDING

This work was funded by Moorfields Eye Charity through a generous donation (A.J.H., M.E.C., P.J.C.), Moorfields Special Trustees (A.J.H., M.E.C., P.J.C.), Wellcome Trust (M.E.C.), MRC/CIRM (P.J.C., L.d.C.), Uren Foundation (P.J.C., L.d.C.) and the Brian Mercer Foundation (P.J.C., L.d.C.). It was also supported by the National Institute for Health Research Biomedical Research Centre at Moorfields Eye Hospital NHS Foundation Trust and UCL Institute of Ophthalmology (A.J.H., L.d.C., P.J.C. are NIHR BRC Faculty). Funding to pay the Open Access publication charges for this article was provided by UCL.

## Supplementary Material

Supplementary Data
