# Supplementary material for: Translational read-through of the RP2 Arg120stop mutation in patient iPSC-derived retinal pigment epithelium cells
Source: Hum Mol Genet. 2014 Oct 6;24(4):972–86. doi: 10.1093/hmg/ddu509 (PMC4986549; doi:10.1093/hmg/ddu509)
Supplement: Supplementary Data [file supp_24_4_972__index.html]

Translational read-through of the RP2 Arg120stop mutation in patient iPSC-derived retinal pigment epithelium cells — Translational read-through of the RP2 Arg120stop mutation in patient iPSC-derived retinal pigment epithelium cells — Supplementary Data 

# Translational read-through of the RP2 Arg120stop mutation in patient iPSC-derived retinal pigment epithelium cells

## Supplementary Data

Supplementary Data

**Files in this Data Supplement:**

- Supplementary Data - Pdf file
