# Supplementary material for: Translational read-through of the RP2 Arg120stop mutation in patient iPSC-derived retinal pigment epithelium cells
Source: Hum Mol Genet. 2014 Oct 6;24(4):972–86. doi: 10.1093/hmg/ddu509 (PMC4986549; doi:10.1093/hmg/ddu509)
Supplement: Supplementary Data [file supp_ddu509_ddu509supp.pdf]

Supplementary materials

Translational read-through of an RP2 Arg120stop mutation in patient iPSC-derived retinal pigment epithelial cells Schwarz, Carr et al

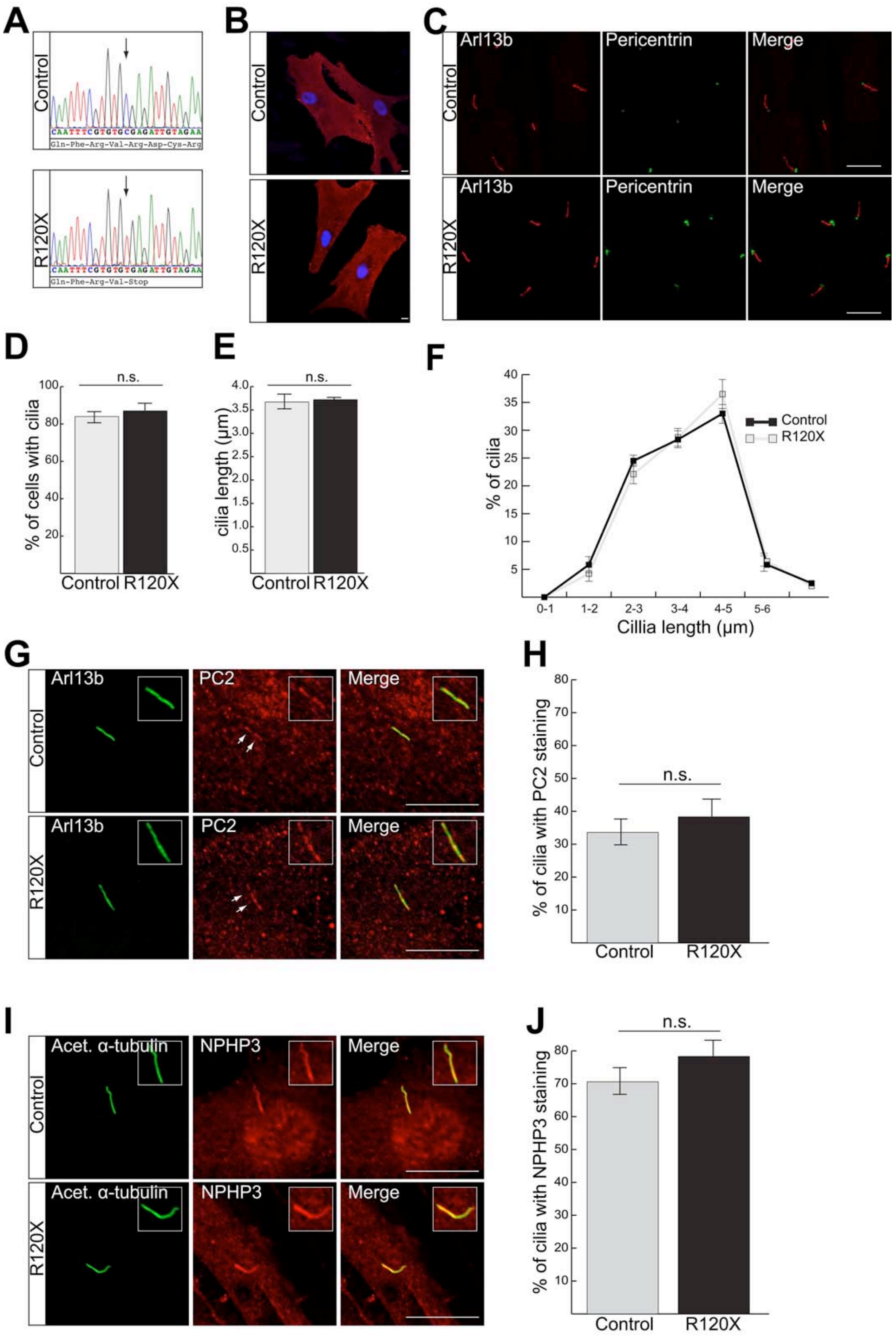

Figure S1. Phenotypic analysis of *RP2* R120X fibroblast cells. (A) Confirmation of the c.519C>T mutation, leading to nonsense mutation R120X in *RP2* patient fibroblasts. **(B)** Immunofluorescence staining of control and R120X fibroblasts with an anti-fibroblast surface protein antibody (red), DAPI (blue), confirming identity of control and patient fibroblasts. Scale bar 10 µm. **(C)** Immunofluorescence staining of fibroblasts with antibodies against the cilia marker Arl13b (green) and the basal body marker pericentrin (red). Scale bar 10 µm. **(D,E,F)** Quantification of cilia incidence (**D**) and length (**E**, **F**) in R120X fibroblasts compared to control cells. **(G)** Immunofluorescence staining of R120X and control fibroblasts for the RP2 interacting protein Polycystin-2 (PC2, red) and the cilia marker Arl13b (green). Cilium is indicated by arrows. The inset shows a higher magnification of the region boxed in the merge image. Scale bar 10 µm. **(H)** Quantification of PC2 staining in R120X and control cilia. Values are means ± 2 SEM, N=300 cells. **(I)** Immunofluorescence staining of R120X and control fibroblasts for the RP2 interacting protein Nephronophthisis 3 (NPHP3, red) and the cilia marker acetylated α-tubulin (green). The inset shows a higher magnification of the region boxed in the merge image. Scale bar 10 µm. **(J)** Quantification of NPHP3 staining in R120X and control cilia. Values are means ± 2 SEM, N=300 cells.

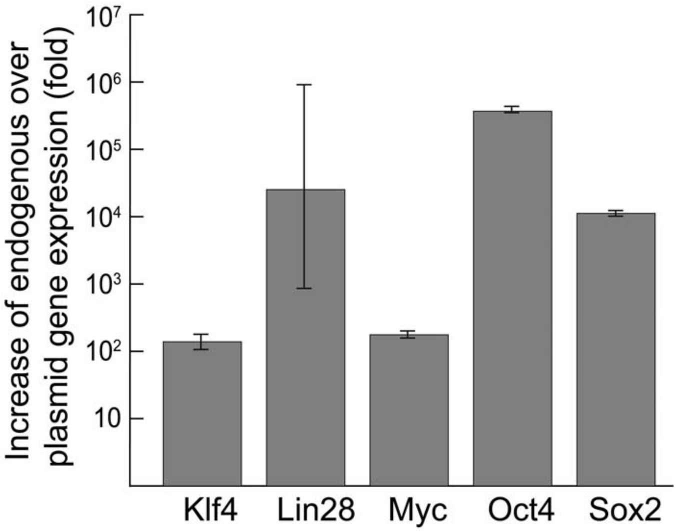

Figure S2. Loss of episomal expression and induction of endogenous pluripotency genes

Analysis of iPSC gene expression in clonal lines by quantitative real-time PCR. RNA was extracted and reverse-transcribed from iPSCs, and using different primer sets in a single PCR reaction, cDNA of endogenous and plasmid transfected Klf4, Lin28, Myc, Oct4 and Sox2 was amplified. Gene expression of endogenous Klf4, Lin28, Myc, Oct4 and Sox2 was then normalised to cDNA amplified from transfected plasmids and shows that endogenous gene expression of these pluripotency markers is highly increased (several hundred to thousand fold) compared to expression from transfected plasmids.

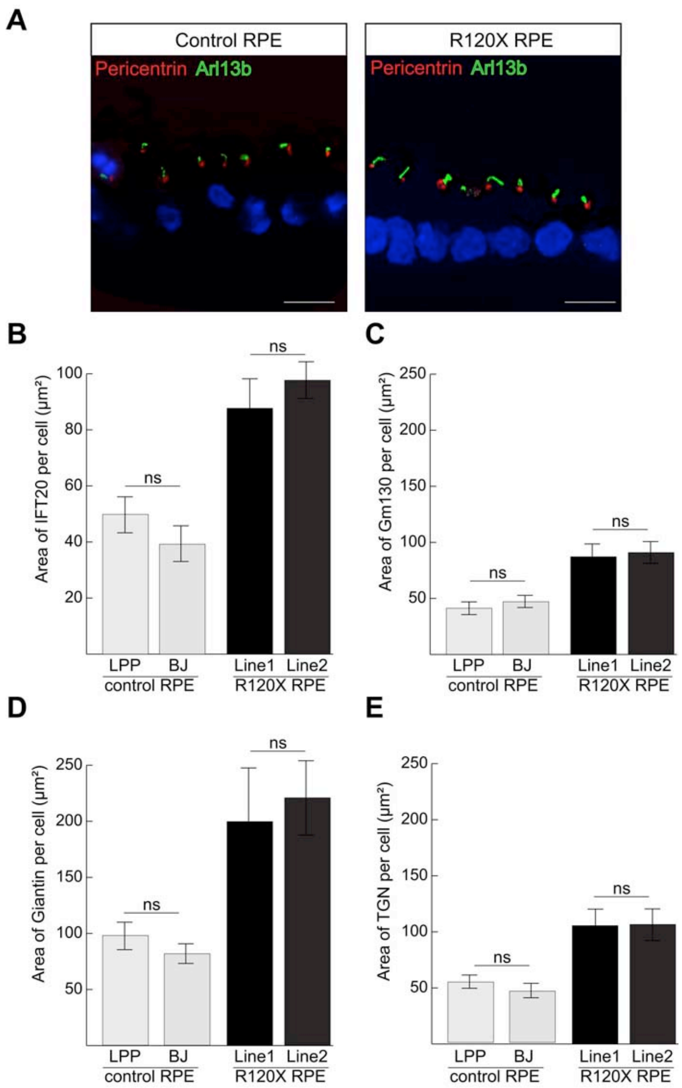

Figure S3. Comparison of independent iPSC-derived patient and control RPE lines. (A) Sectioned RPE cells were stained for the cilia marker Arl13b (green) and the basal body marker pericentrin (red), DAPI (blue). Cilia expression in the R120X RPE cells is comparable to control RPE cells. Scale bar 10 µm. (**B**) Two independent control iPSC-derived RPE lines from two different males (LPP and BJ) and two independent iPSC-derived R120X RPE lines, Line 1 and Line 2, were analysed for IFT20 dispersal and quantified as described. IFT20 was dispersed in both R120X lines, with no significant difference between the two lines. IFT20 staining was

comparable in both control RPE lines. Values are means ± 2 SEM, N=300 cells. **(C, D, E)** Similarly, Golgi morphology (**C**; GM130, **D**; Giantin and **E**; TGN) was assessed in the two independent iPSC-derived R120X RPE (Line 1 and Line 2) and control RPE (LPP and BJ) lines. Golgi cohesion was disrupted in both R120X lines, with no significant difference between the two lines. The control RPE lines (LPP and BJ) showed no difference in Golgi cohesion for the analysed markers. Values are means ± 2 SEM, N=300 cells.

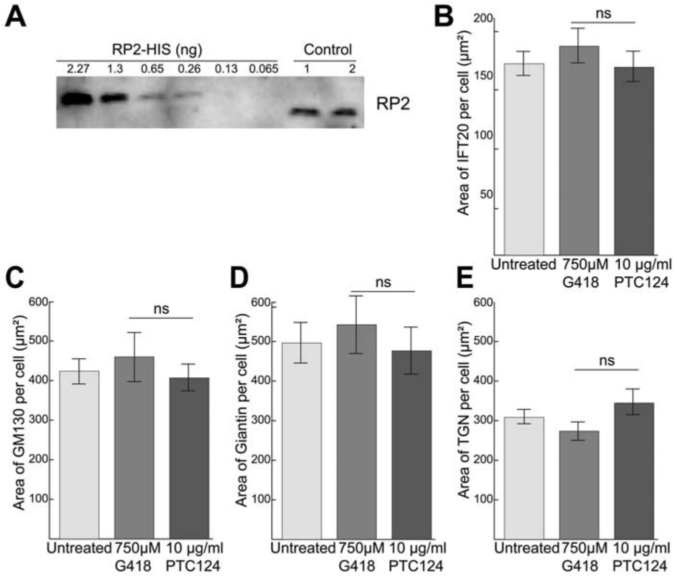

Figure S4. RP2 calibration and lack of off-target effects of TRIDs in control male fibroblasts. (A) Quantification of RP2 protein levels from two independent whole cell lysates in control fibroblasts by Western blotting. R120X fibroblast whole cell lysates were mixed with known concentrations of recombinant RP2-HIS protein to establish a titration sensitivity curve, and run alongside control lysates. RP2 concentration in control fibroblasts was quantified using ImageJ software. **(B)** Analysis of potential off-target effects of G148 and PTC124 in control male fibroblast cells. Control fibroblasts were treated with a single 24-hour dose of 750 µM G418 or 10 µg/ml PTC124, and IFT20 area was measured as described previously. Treatment with either drug did not affect IFT20 localisation. Values are means ± 2 SEM, N=300 cells. **(C, D, E)** G418 and PTC124 show no effect on Golgi morphology or area. Control cells were treated as above and stained for the cis, cis-medial and trans Golgi markers GM130, Giantin and TGN. Effect on Golgi area was measured as described. Values are means ± 2 SEM, N=300 cells.
